# Effect of Tai Chi combined with visual-action-sensory rehabilitation therapies on cognitive function in acute ischemic stroke: a study protocol for a randomized controlled trial

**DOI:** 10.1080/07853890.2025.2566877

**Published:** 2025-10-06

**Authors:** Xuewei Guan, Meijuan Lan, Lan Ge, Qianyin Zhu, Yuanyuan Chen, Leiwen Tang, Yumei Zhong

**Affiliations:** Nursing Department, the Second Affiliated Hospital of Zhejiang University School of Medicine, Hangzhou, China

**Keywords:** Acute ischemic stroke, mirror neurons, action observation therapy, sensory observation therapy, cognitive rehabilitation

## Abstract

**Introduction:**

Early cognitive intervention in patients with acute ischemic stroke (AIS) is associated with better outcomes. ‘Visual-action-sensory’ rehabilitation therapies, including action observation therapy (AOT) and sensory observation therapy (SOT) have shown great potential in restoring cognitive function. A systematic review of some studies on the therapeutic effect of Tai Chi (TC)in stroke patients suggests potential benefits on depression, anxiety, and balance function. Therefore, combining AOT with TC may enhance brain functional connectivity and provide some cognitive improvements. Here, we describe a study protocol assessing the long-term effects of TC-AOT combined with SOT on cognitive function with AIS.

**Methods:**

This study is a dual-arm, single-center, single-blind, randomized controlled trial. A total of 86 AIS patients will be enrolled and randomly assigned in a 1:1 ratio to either the intervention group or the control group. The control group will receive conventional exercise education and follow-up. The intervention group first watched tactile stimulation followed by TC action videos, and then practiced imitating TC movements. The program will be implemented once a day for 30 min, 5 days a week for a duration of 8 weeks. Outcome measures will be assessed at baseline, week 8, and week 12. The primary outcome is global cognitive function and secondary outcomes are language, attention, executive function, memory, visuospatial ability, neuropsychological assessments, and quality of daily life.

**Conclusion:**

The study anticipates that the therapeutic program described here will help to reduce the incidence of cognitive impairment in AIS patients or delay its progression.

**Trial registration:**

We have registered at https://www.chictr.org.cn and the registration number is: ChiCTR2400088156.

## Administrative information

This paper is based on protocol version 2.0(2024-07-24). Trial sponsor: Meijuan Lan.

## Introduction

Stroke is the second leading cause of death and the third leading cause of disability worldwide [1]. Acute ischemic stroke (AIS), the most common type, accounted for 69.6%∼72.8% of cases in China [2,3]. At the acute stage, cognitive decline prevalence in ischemic stroke was 88.1%, and this impairment is linked to a higher risk of future disability [4]. To date, there are no proven effective treatments to reverse post-stroke cognitive impairment (PSCI) [5]. Therefore, it is crucial to initiate interventions at the preclinical and early stages to prevent and delay the onset of PSCI, as individuals at this stage still have the capacity to meaningfully engage in interventions [6]. At present, the non-pharmacological approach remains the main strategy to preserve cognitive functions in populations with cognitive decline [7]. Among these non-pharmacological interventions, action observation therapy (AOT) and sensory observation therapy (SOT) have shown great potential in restoring cognitive function.

AOT and SOT are key components of ‘visual-action-sensory’ rehabilitation therapy. In AOT, participants watch others performing goal-directed movements. This visual input activates motor-related brain regions responsible for planning and executing similar actions in the observer [8]. In SOT, participants observe others touching various textured materials. This process evokes internal simulation of the tactile experience, engaging somatosensory areas as if the participants themselves were feeling the stimuli [9]. The activation of these sensorimotor responses is mediated by the mirror neuron system (MNS). Mirror neurons are primarily located in the inferior parietal lobule, the ventral premotor cortex, and the caudal part of the inferior frontal gyrus [10]. These areas overlap with regions involved in cognitive functions. Through this widespread network, the MNS allows the brain to internally simulate observed actions or sensations, engaging corresponding functional areas as if the individual were performing the action or experiencing the stimulus. MNS is closely related to processes such as environmental information processing and sensory selection [11,12]. Thus, mirror neurons engage in complex cognitive processes by simulating observed content through visual input, facilitating the formation and strengthening of neural circuits. This can lead to the creation of new synaptic connections or the reinforcement of existing ones [13,14]. By forming new synapses, these therapies adjust and optimize learning and memory processes, thereby improving the efficiency of information processing and storage to compensate for cognitive impairments in stroke patients [15].

Previous systematic reviews have shown that AOT may benefit global cognitive function and language function in various populations [16,17]. However, we found that in most existing studies, participants were exposed to AOT videos that were fragmented and randomly presented, lacking systematic structure and continuity. To address this limitation, we adopted Tai Chi-based action observation therapy (TC-AOT), which uses videos of traditional Chinese Tai Chi routines characterized by spiralling motion and sequential coordination. Tai Chi movements are not only culturally familiar and widely accepted in China, but also support visuospatial processing, processing speed, and episodic memory [18]. Studies have shown that after TC-AOT intervention, there is a significant increase in brain functional connectivity between areas involved in executive attention and motor learning, including the temporal parietal junction (TPJ), supramarginal gyrus (SMG), inferior frontal gyrus (IFG), and insula [19]. The modulation of TPJ and SMG can influence motor attention in complex skilled actions [20,21], the IFG is crucial for controlling complex motor actions [22], and the insula integrates sensory information for motor planning [23]. Thus, TC-AOT holds significant potential benefits for cognitive rehabilitation.

SOT uses visual input to observe tactile sensory information in videos, enhancing patients’ information processing abilities, thereby promoting brain function recovery and cognitive improvement [9,24]. The mirror neuron system simulates sensory experiences; The mirror neuron system simulates sensory experiences; observing someone touch a material prompts the brain to recognize the action and partially simulates the tactile experience [9]. This understanding and prediction of others’ sensory states represent a highly complex cognitive function [25,26]. Therefore, it can be inferred that sensory observation activates sensory regions of the mirror neuron system, forming sensory representations that further enhance the brain’s sensory information processing and integration, potentially improving cognitive function.

Research has found that engaging in sensory observation before watching actions can more effectively improve cognitive function. This method’s effectiveness stems from deeply utilizing of the brain’s information-processing mechanisms [27]. Sensory observation, as an initial step, helps activate sensory processing areas in the brain, providing a strengthened neural foundation for subsequent action observation [28]. Once the brain is “primed” in sensory information processing, observing and simulating actions may be more profound and effective, promoting broader neural network activation and reorganization, further enhancing cognitive recovery and improvement. This finding highlights the importance of integrating sensory and action observation in cognitive intervention strategies.

Although some studies on the therapeutic effect of Tai Chi in stroke patients suggest potential benefits on depression, anxiety [29], and balance function [30], an evaluation of the cognitive benefits of Tai Chi in combination with other visual-motor-sensory’ rehabilitation therapies have received scanty attention. Therefore, this study combined TC-AOT with SOT to evaluate its cognitive effects on AIS patients. The results of this study will provide useful insights into the therapeutic value of combining AOT with Tai Chi to enhance brain functional connectivity and provide cognitive improvements.

## Patients and methods

### Study design and setting

**Figure 1.**
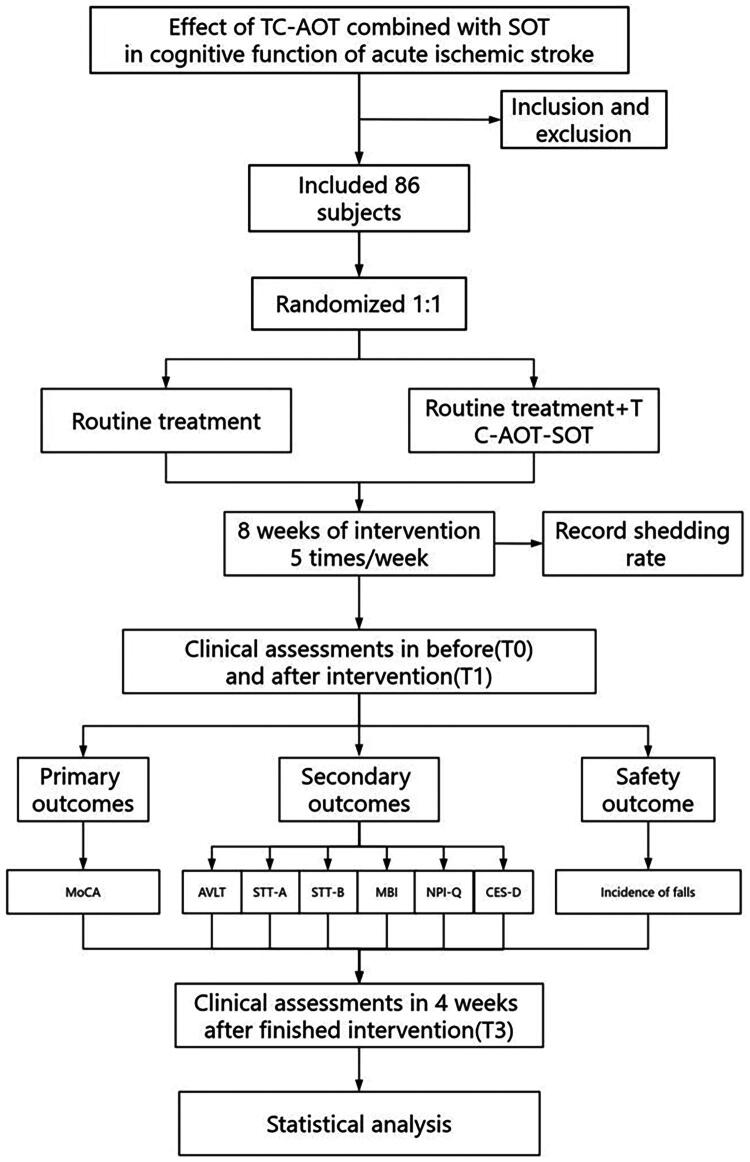
Flowchart of the trial design.

This is a 2-group parallel, single-site, single-blind, hospital and home-based randomized controlled trial. Patients with AIS will be recruited from a Hospital in Zhejiang Province. The study consists of an 8-week intervention and a 4-week follow-up (Figure 1). All patients will provide written informed consent in accordance with the 2018 version of the Declaration of Helsinki.

### Eligibility criteria

#### Inclusion criteria

1. The first stroke was in accordance with the Chinese guidelines for diagnosis and treatment of acute ischemic stroke 2023 [31] and was confirmed by brain CT or MRI.
2. First onset of stroke, with disease duration of less than 14 days;
3. The NIHSS score less than 5;
4. Muscle strength of limbs > level 3.

#### Exclusion criteria

1. Patients currently participating in other cognitive interventions;
2. Patients suspected of cognitive impairment before stroke (screening with AD8);
3. Significant aphasia or severe cognitive impairment, inability to understand or cooperate with assessment and treatment instructions;
4. Complicated with severe cardiopulmonary diseases, mental disorders, multiple organ failure, malignant tumors, or seizures.

### Sample size calculation

Calculate the sample size according to the following formula:

$$n=2{\left(\mu_{\alpha }+\mu_{\beta }\right)}^{2}\sigma^{2}/\delta^{2}$$

*n* is the sample size for each group; *μ_α_* is the u value corresponding to the Type I error probability, and *μ_β_* is the u value corresponding to the Type II error probability; *δ* donates the expected effect size, i.e. the absolute value of the difference in means between the two groups, and σ^2^ is the population variance, estimated by the sample variance, which is the mean of the variances of the two groups. *α* is set at 0.05 and *β* is set at 0.10. According to Yeh et al.’s study [32], σ = 4.985, δ = 2.33, the calculated sample size for each group is 30. Considering a 25% dropout rate in previous studies [33] and the potential for a higher dropout rate in cognition-related studies [34], we increased the dropout rate to 30%. Ultimately, we determined that each group should include 43 patients.

### Recruitment

Potential participants in the hospital will be identified by screening the electronic medical records in the neurology ward to find patients who experienced their first ischemic stroke within 14 days of onset. A research coordinator will then contact these potential participants to assess their eligibility, ability to complete the questionnaire, interest in participation, experience using WeChat, and willingness to sign the consent form along with one of their family members.

### Randomization and blinding

The statistician will use SPSS 26.0 software to randomize the sequence numbered 1–86 using a random number generator. Random numbers for grouping will be generated and divided into two groups in a 1:1 ratio using visual binning. The grouping results are placed in opaque envelopes. Eligible participants will receive an envelope in registration order and hand it over to the intervention implementer. The researcher who sealed the envelopes will not be involved in participant inclusion, treatment, or assessment. Due to the nature of action-sensory observation, blinding participants and intervention implementers is not possible. However, participants will receive individual treatment, and the intervention implementers will not participate in outcome assessments. Evaluators and statisticians will remain unaware of group assignments.

### Interventions

**Figure 2.**
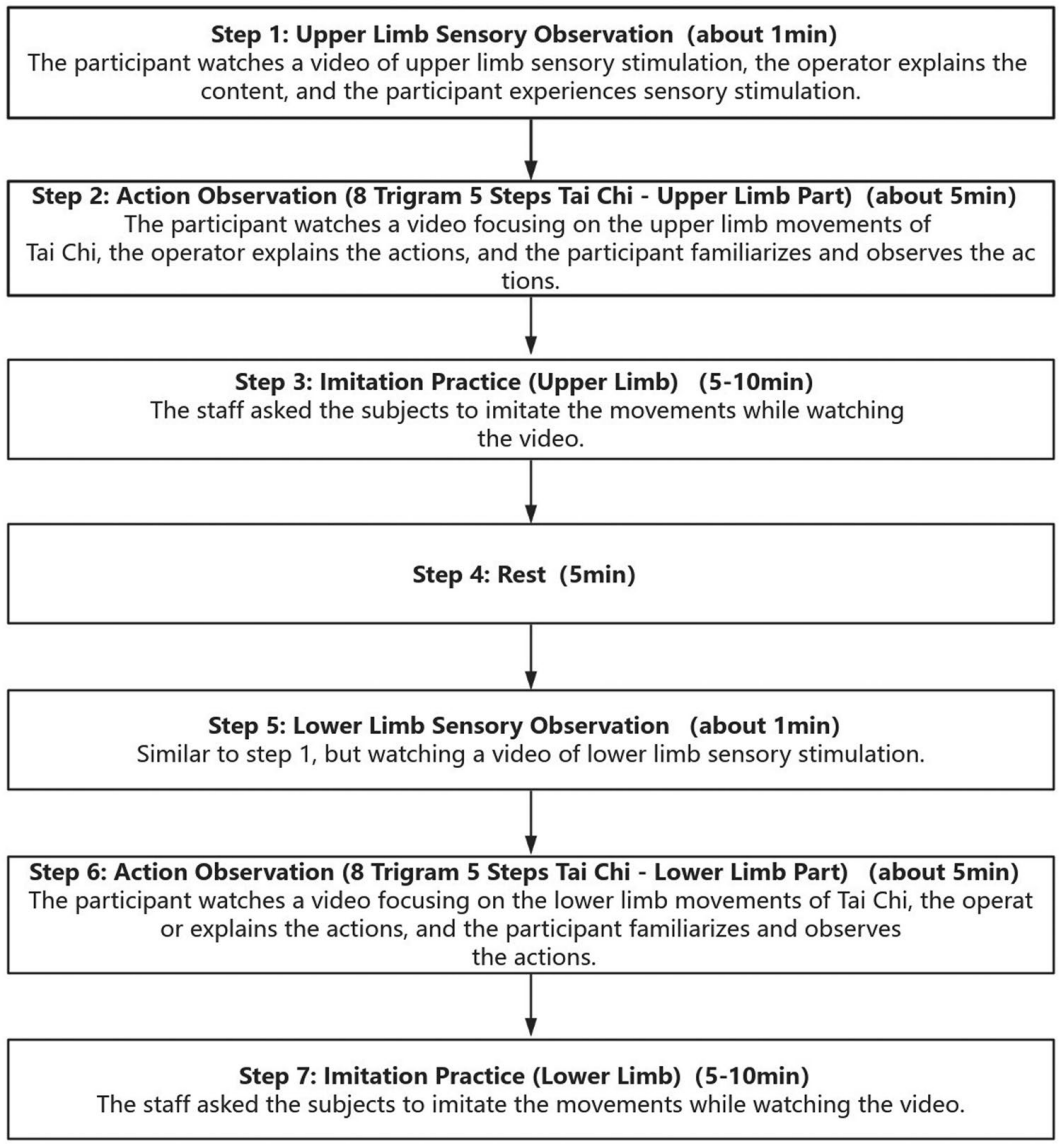
TC-AOT combined with SOT therapy flow chat.

In this prospective, two-arm, single-centre, randomized controlled trial study, we will recruit 86 acute ischemic stroke patients within 14 days of onset. Participants will be randomly assigned to either an intervention group (receiving TC-AOT combined with SOT) or a control group (undergoing routine cognitive rehabilitation) in a 1:1 ratio. All patients will receive routine treatment and conventional exercise education, delivered by professional therapists during hospitalization. After discharge, education and follow-up will be conducted by our medical and nursing team. Additionally, in the intervention group, patients will begin by watching a sensory activation video, followed by watching a video on 8 Trigram 5 Steps TC, and then engaging in TC exercises. The program will be implemented once a day for 30 min, 5 days a week, and will last for 8 weeks. Based on the results of the preliminary literature review and evidence-based analysis, and after an expert panel meeting, the interventions were ultimately developed as illustrated in Figure 2.

The AOT video selects the 8 Trigram 5 Steps TC released by the General Administration of Sports of China, and the sensory observation video was recorded by the researchers ourselves. The sensory observation for both the upper and lower limbs involved quick tapping with a wooden board (20s), brushing with silk (20s), and brushing with a cotton swab (20s).

In the hospital, the training will be conducted in the neurology ward. The intervention schedule will run from Monday to Friday, with one session in the morning (10:30–11:00) and three sessions in the afternoon (15:00–15:30, 15:45–16:15, and 16:30–17:00). Patients will be required to participate in only one session per day. After discharge, the researchers will send the videos to the patients and provide daily reminders from Monday to Friday to watch and imitate the videos. During the home-based intervention, a caregiver will accompany the patient throughout the entire video-guided training session and take three photos capturing the patient actively engaged in the intervention. These photos will be uploaded *via* the “Jielong Butler” WeChat applet as evidence of participation. Participants are required to complete this check-in five times per week. The total duration of the intervention will be 8 weeks.

### Outcomes

At baseline, patients will undergo the AD8 test to screen for pre-stroke cognitive impairment; those identified with such impairment will be excluded. Routine sociodemographic data will also be collected, including age, gender, marital/cohabitation status, employment status, as well as personal and family medical history, and smoking and alcohol consumption habits.

Validated assessment tools will be employed to evaluate global cognitive function, memory, language function, executive function, attention, visuospatial ability, activities of daily living, neuropsychiatric symptoms, and psychological status. Each scale will be analysed independently, no composite total score will be calculated across different scales.

Subjects will be evaluated at three time points: before the intervention (1–2 days before the start, T1), after the intervention (1–2 days following the 8-week intervention, T2), and during follow-up (4 weeks after the 8-week intervention, T3). The safety outcome, particularly the incidence of falls, will be monitored throughout the study.

### Primary outcomes

Montreal Cognitive Assessment (MoCA) [35] assesses visuospatial and executive function, naming, memory, attention and computation, language, abstract thinking, delayed recall, and orientation. The test comprises 30 questions and the highest possible score is 30. The sensitivity of MoCA is 92.4%, the specificity is 88.4%, the best abnormal cut-off point is: illiterate group ≤ 13 points, primary school group ≤ 19 points, junior high school, and above group ≤ 24 points.

### Secondary outcomes

Auditory Verbal Learning Test (AVLT) is one of the most commonly used memory assessment scales. In this study, the Chinese version of AVLT developed by Guo Qihao et al. [36] was adopted. The scale uses 12 words. During the test, the examiner reads the 12 words aloud, and participants are asked to recall them immediately. They continuously learn and recall the words three times, with reminders provided beforehand. After an approximately 5-minute non-verbal test interval, participants perform “short delayed recall” of the words, and after a 20-minute interval, they perform ‘long delayed recall’. The sixth recall is re-recognition, where the examiner presents 24 words and asked the subjects to identify which were learned.

The Animal Fluency Test (AFT) is used to assess a patient’s language function [37]. Participants list as many animals as possible within 60 s, with each correctly named animal counting as one point. A higher score indicates better language function.

The Clock Drawing Test (CDT) primarily assesses a patient’s visuospatial function. Participants draw a clock on a blank sheet, adding numbers and setting the hands to 11:10. The scoring method follows the Rouleau 10-point scale: clock face integrity (maximum 2 points), presence and sequencing of numbers (maximum 4 points), and presence and placement of the hands (maximum 4 points) [38].

The Shape Trails Test (STT) was developed by the U.S. Army in 1944 as part of the Halstead-Reita suite of neuropsychological tests. This study uses a version by Guo Qihao et al. [39]. The test has two parts, A and B, involving two shapes: square and circle. STT-A requires connecting numbers, assessing attention and processing speed with a maximum of 150 s. STT-B involves alternating connections of shapes, evaluating executive function with a maximum of 300 s. For both tests, shorter completion times indicate better performance.

The Modified Barthel Index (MBI) [40] assesses activities of daily living (ADLs), covering ten areas: feeding, dressing, toileting, personal hygiene, bathing, bed-chair transfer, walking, stair climbing, bowel control, and bladder control. A score of ≤40 indicates severe dependency, 41 to 60 implies moderate dependency, 61 to 99 reflects mild dependency, and 100 signifies normal activity without assistance.

The Neuropsychiatric Inventory-Questionnaire (NPI-Q) is used to assess neuropsychiatric symptoms [41]. It consists of 10 behavioral and 2 autonomic nervous system symptom domains. Researchers gather information about symptoms over the past month from caregivers. Symptoms are marked “present” if they occurred and rated for severity (0–36 points) and caregiver distress (0–60 points). The Chinese version of the scale has a Cronbach’s α of 0.85, split-half reliability of 0.83, and test-retest reliability of 0.86 [42].

This study uses the short form of the Center for Epidemiologic Studies Depression Scale (CES-D), comprising 9 items: 2 positive and 7 negative symptoms. Internal consistency reliability ranges from 0.85 to 0.88. Scoring reverses positive symptoms, with a total of 27 possible points. A score of 10 indicates a tendency toward depression, while 17 marks the high-risk threshold [43].

### Safety outcomes

Fall is a sudden, involuntary, or unintentional fall to the ground or other lower surface not caused by a violent blow, loss of consciousness, sudden paralysis, or seizure [44]. Fall rates are usually expressed per 1,000 bed days. In rehabilitation hospitals where patients are encouraged to mobilize, fall rates typically range from 3 to 16 per 1,000 bed days [45].

For an overview of the study schedule, see Table 1.

**Table 1.** Schedule of enrolment, interventions and assessments according to the standard protocol items: Recommendations for interventional Trials (SPIRIT).

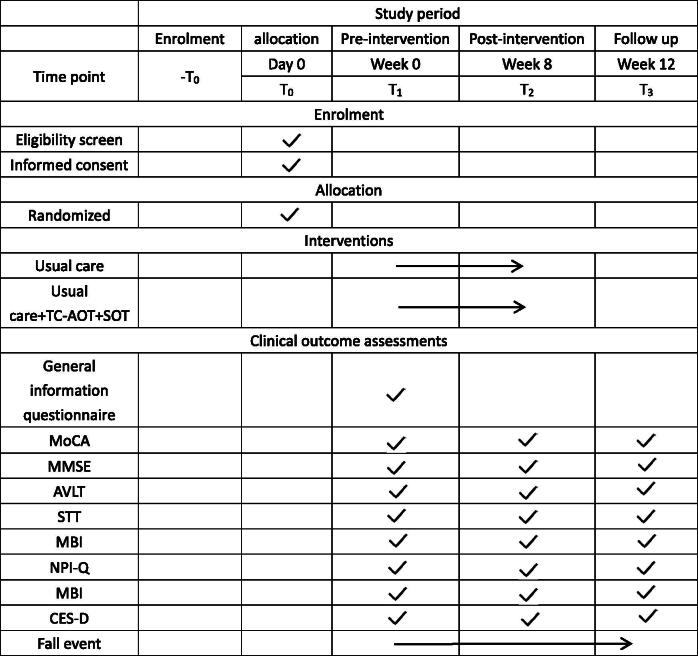					

### Data collection and management

After patients sign the written informed consent form, scale assessments will be conducted. Each patient will have a separate folder to store their informed consent forms and completed scales. All the clinical data will be input into EpiData3.1 by two trained research assistants independently. Only the research team members have the right to access. All researchers will be trained regularly, and all data will be monitored and verified regularly by investigators throughout the trial.Due to the trial’s short duration and minimal risks, a Data Monitoring Committee will not be established. Data can be obtained by contacting the tutor after the RCT is published.

### Data analysis

A statistician will analyse the data using SPSS 26.0 software, considering a P-value < 0.05 as statistically significant. Continuous variables will be presented as mean ± standard deviation or median with interquartile range, while categorical variables will be expressed as frequency and percentage. The outcome measures in this study are all continuous variables. Differences in outcomes at baseline, week 8, and week 12 will be analysed using repeated measures ANOVA or mixed linear regression analysis. Both the intention-to-treat (ITT) and per-protocol (PP) populations will be included in the analysis. The ITT and PP analysis results will be compared to ensure consistency. In cases of missing data, the last observed value will be carried forward for interpolation. A safety analysis will be performed to assess safety outcomes.

### Monitoring

To enhance patient adherence and ensure the effectiveness of the trial, researchers will establish a rapport with patients during communication, inquiring about their level of enthusiasm for participation. Patients will be informed that after discharge, they will need to watch the training videos at home and check in using the ‘Jielong Butler’ WeChat applet. To verify the authenticity of each training session, patients will be required to upload three photos showing their ongoing training activities. These photos will be submitted by the patients’ family members and reviewed by the research team to ensure compliance. This process will also ensure that patients are accompanied by their family members during the exercises. Additionally, patients who consistently complete the check-ins for 8 weeks will receive a small gift as an incentive. After these details are communicated to the patients and their families, the written informed consent will be obtained, and the contact information of the patients and their families will be recorded for follow-up and monitoring.

Data collectors received standardized training on questionnaire evaluation, mastering the content, assessment methods, and techniques while remaining unaware of the study’s group assignments. During data collection, they refrain from guiding or suggesting responses to participants, and the intervention will not be mentioned during assessments to maintain objectivity and consistency. Two university researchers are responsible for data entry and verification to ensure accuracy.

Although the intervention is non-invasive, adverse events such as falls or fractures may occur. To ensure safety, patients and their families will be provided with staff contact information *via* phone or WeChat, and instructed to contact staff immediately if any adverse events occur. Details of all adverse events will be recorded during the trial in the case report forms (CRFs). The ethics committee will examine any connections between adverse events and the intervention and decide whether the study should continue.

## Ethics and dissemination

The present study protocol has been approved by the Regional Ethics Authority of the Second Affiliated Hospital of Zhejiang University School of Medicine (Grant No.2024-0591). This protocol adheres to the Standard Protocol Items: Recommendations for Clinical Interventional Trials (SPIRIT) guidelines for clinical interventional trials. Researchers will communicate with eligible participants, fully explain the study procedures, and obtain informed the written consent from those willing to participate. Participant data will be recorded in CRFs. If the researchers need to get the data in the CRF, they must report to the primary investigator.

## Discussion

Cognitive impairment is a common post-stroke symptom among recurrent AIS patients, associated with reduced functional independence, institutionalization, diminished quality of life, and and increased mortality [46,47]. Thus, stroke survivors need interventions to promote cognitive function and prevent dementia. ‘Visual-action-sensory’ rehabilitation therapies, including AOT and SOT, have shown great potential in restoring cognitive function. During action and sensory observation, the brain achieves functional reorganization through the coordinated action of the mirror neuron system. In action observation, the superior temporal sulcus encodes and decodes action information, transmitting it to the parietal and frontal mirror neuron systems to facilitate the understanding of actions and intentions [48]. In sensory observation, the activated precentral gyrus and supplementary motor area, as components of the parietal-frontal mirror system [49], are responsible for motor execution and preparation, while the postcentral gyrus integrates sensory input [50,51]. These regions work together through mirror neuron mechanisms to enhance understanding and imitation of others’ actions and sensory experiences.

The intervention designed in this study represents an enhancement and refinement of traditional ‘visual-action-sensory’ rehabilitation therapies. In this study, the content of action observation was modified to TC, as TC-AOT has been shown to provide more comprehensive cognitive improvement by enhancing functional connectivity between the dorsomedial prefrontal cortex and the cerebellar Crus I, as well as the parietal-occipital lobe and other regions [19]. These connections support more complex brain coordination and integration capabilities. In contrast, traditional AOT mainly activates the premotor cortex and certain parietal and temporal lobe regions, making it less comprehensive than TC-AOT in integrating cognitive and motor coordination [52]. Therefore, TC-AOT may be more advantageous in promoting a broader reorganization of brain function. Moreover, this study is the first to apply SOT in a clinical setting. In patients with sensory disorders, somatosensory stimuli cannot be effectively transmitted to the cerebral cortex [53], leading to the loss of sensory part in the ‘visual-motion-sensation’ therapy. To allow patients with impaired sensory pathways to benefit from sensory stimulation, we introduced sensory observation [54]. This approach allows the activation of patients’ sensory circuits without actual sensory input, achieving effects similar to normal sensory stimulation. The TC-AOT combine with SOT scheme designed in this study not only adopts the more effective motion observation (TC-AOT), but also fills the gap that somatosensory information cannot be transmitted in patients with sensory disorders through sensory observation. This dual innovation may enhance the effectiveness of the intervention.

The brain’s ability to reorganize itself, known as neuroplasticity, peaks immediately after stroke [55]. Timely and appropriate training and stimulation allow undamaged neurons to reconfigure their connections, compensating for functions of damaged regions and optimizing rehabilitation outcomes [56]. Existing research indicates that implementing non-routine or alternative therapies within 1 to 60 days post-stroke results in better cognitive recovery outcomes compared to homologous interventions during the chronic phase [57]. Thus, this study advances the intervention window to capitalize on the nervous system’s heightened plasticity. By administering precise treatment and stimulation during this acute period, we aim to more effectively facilitate brain reorganization and functional recovery, thereby preventing cognitive impairments or enhancing cognitive function in patients with deficits.

We anticipate that the therapeutic program described here will help AIS patients prevent the onset of cognitive impairment and improve their overall quality of life. The planned randomized controlled trial will contribute evidence on the effectiveness of integrating TC-AOT and SOT in stroke rehabilitation.

However, implementing the therapy may pose challenges such as prolonged intervention cycle and poor patient adherence. Additionally, this study is single-centered, which may limit the generalizability of this approach to other centers. Another limitation lies in participants in the intervention group received longer exposure time, which could bias the results. Furthermore, future studies should consider incorporating functional connectivity biomarkers (e.g. fNIRS, EEG) to provide more objective insights into the neural mechanisms of cognitive recovery. Multidisciplinary collaboration will be essential to achieve this goal and further advance the field of stroke rehabilitation.

## Conclusion

The lack of early preventive interventions for AIS patients in clinical practice necessitates the need to develop viable evidence-based intervention programs. The protocol of RCT seeks to achieve this end. If effective and verified by follow-up studies, the TC-AOT combined with SOT may help reduce the incidence of cognitive impairments in AIS patients or delay the clinical progression of such impairments.

## Supplementary Material

SPIRIT checklist with page reference numbers.doc

## Data Availability

After formal publication of this trial, anonymized data will be made available upon request. For access, please contact the corresponding author, Meijuan Lan.
